# Surface-modified nanoparticles as anti-biofilm filler for dental polymers

**DOI:** 10.1371/journal.pone.0189397

**Published:** 2017-12-15

**Authors:** Nathan Zaltsman, Andrei C. Ionescu, Ervin I. Weiss, Eugenio Brambilla, Shaul Beyth, Nurit Beyth

**Affiliations:** 1 Department of Prosthodontics, Hebrew University-Hadassah School of Dental Medicine, Jerusalem, Israel; 2 Department of Biomedical, Surgical and Dental Sciences, IRCCS Galeazzi Institute, University of Milan, Milan, Italy; 3 Goldschleger School of Dental Medicine, Tel Aviv University, Tel Aviv, Israel; 4 Orthopedic Surgery Complex, Hadassah University Hospital, Jerusalem, Israel; Institute of Materials Science, GERMANY

## Abstract

The objective of the study was to synthesis silica nanoparticles modified with (i) a tertiary amine bearing two t-cinnamaldehyde substituents or (ii) dimethyl-octyl ammonium, alongside the well-studied quaternary ammonium polyethyleneimine nanoparticles. These were to be evaluated for their chemical and mechanical properties, as well for antibacterial and antibiofilm activity. Samples were incorporated in commercial dental resin material and the degree of monomer conversion, mechanical strength, and water contact angle were tested to characterize the effect of the nanoparticles on resin material. Antibacterial activity was evaluated with the direct contact test and the biofilm inhibition test against *Streptococcus mutans*. Addition of cinnamaldehyde-modified particles preserved the degree of conversion and compressive strength of the base material and increased surface hydrophobicity. Quaternary ammonium functional groups led to a decrease in the degree of conversion and to low compressive strength, without altering the hydrophilic nature of the base material. In the direct contact test and the anti-biofilm test, the polyethyleneimine particles exhibited the strongest antibacterial effect. The cinnamaldehyde-modified particles displayed antibiofilm activity, silica particles with quaternary ammonium were ineffective. Immobilization of t-cinnamaldehyde onto a solid surface via amine linkers provided a better alternative to the well-known quaternary ammonium bactericides.

## Introduction

Contact-acting non-leachable antibacterial compounds are drawing increased attention in recent years. They may be regarded as a promising solution for contamination of medical devices surfaces, including dental restorations. Several approaches are used to incorporate antibacterial agents such as quaternary ammonium salts (QAS) in dental resin-based materials. For example, the acrylic monomer 12-methacryloyloxydodecylpyridinium bromide (MDPB) [1,2] has a methacrylate group available for co-polymerization with the host resin and a pyridinium cation linked to a long alkyl chain spacer. Similar acrylic monomers, modified with alkyl-dimethyl QAS and of different alkyl chain length, were reported to have antibacterial properties [1]. Another proposed variation of QAS-containing acrylates includes dimethacrylates such as ionic dimethacrylate monomers [2], with dimethylammonium in the backbone. Aside from the antibacterial monomers added to the resin matrix, antibacterial solid nanoparticles (NPs) with QAS functionality were proposed. Quaternary ammonium polyethyleneimine (QPEI) nanoparticles were found to be contact-active bactericides when incorporated in various resin-based materials [3–11]. These are macromoleculular nanoparticles, i.e. the cross-linked, high molecular weight polyethyleneimine forms an NP core that is further functionalized with dimethyl-octyl-ammonium QAS. As QPEI are prepared mainly from polyethyleneimine, these NPs have an amine content of extremely high density, which is reflected by a strong positive charge [5]. However, the excess iodine that remained attached to these nanoparticles after their preparation can inhibit free-radical polymerization of resin-based materials [12]. This inevitably leads to a low degree of monomer conversion, and thus reduced mechanical properties, with monomer leaking out of the composition.

Silica dioxide NPs could be a better alternative as core material instead of polyethyleneimine because they do not involve iodide-containing reagents during their preparation. Size-controlled preparation of silica NPs, introduced by Stober [13], gained much interest in the biomaterials field, as they could be prepared in variable size and degree of functionality loading [14–17]. In addition to the previously described and well-known QAS-based antibacterial agent, tertiary amine polycation fiber, namely dimethyl amine polystyrene [18] was reported to have a contact-active antibacterial effect. Substitution of amine groups possessing methyl functionality by well-known antibacterial molecules of natural source may yield new efficient surface-active bactericides. Potent bactericides might be present in essential oils like trans-cinnamaldehyde (t-cial), an extracted aldehyde from the genus *Cinnamomum* [19–23]. Moreover, modification of t-cial with amino-functionalities, such as amino acids [24], produced imine thiosemicarbazones (Schiff bases) [25], which too exhibited antibacterial activity. Nevertheless, the exact antibacterial mechanism of t-cial remains unknown, although it is considered to affect the bacterial membrane, as well as interfere in its metabolism [26]. Although t-cial is a known bactericide when used as a free molecule in solution or in controlled release, there is no evidence that it preserves its antibacterial properties when irreversibly immobilized on a solid surface.

The new antibacterial particles proposed herein have a lower number of amine functional groups and a chemically inert core. The t-cinnamaldehyde molecule was immobilized on amino-functionalized silica nanoparticles to assess whether it preserves its antibacterial activity when added to a dental resin-based material. To compare the antibacterial effect of immobilized t-cial, also amino-functionalized silica NPs were prepared with the *N*-octyl, *N*,*N*-dimethyl functional group, as previously shown for QPEI [3]. Physical characteristics such as contact angle, mechanical strength, degree of monomer conversion and discoloration were also investigated.

## Materials and methods

### Silica NP preparation

**Fig 1:**
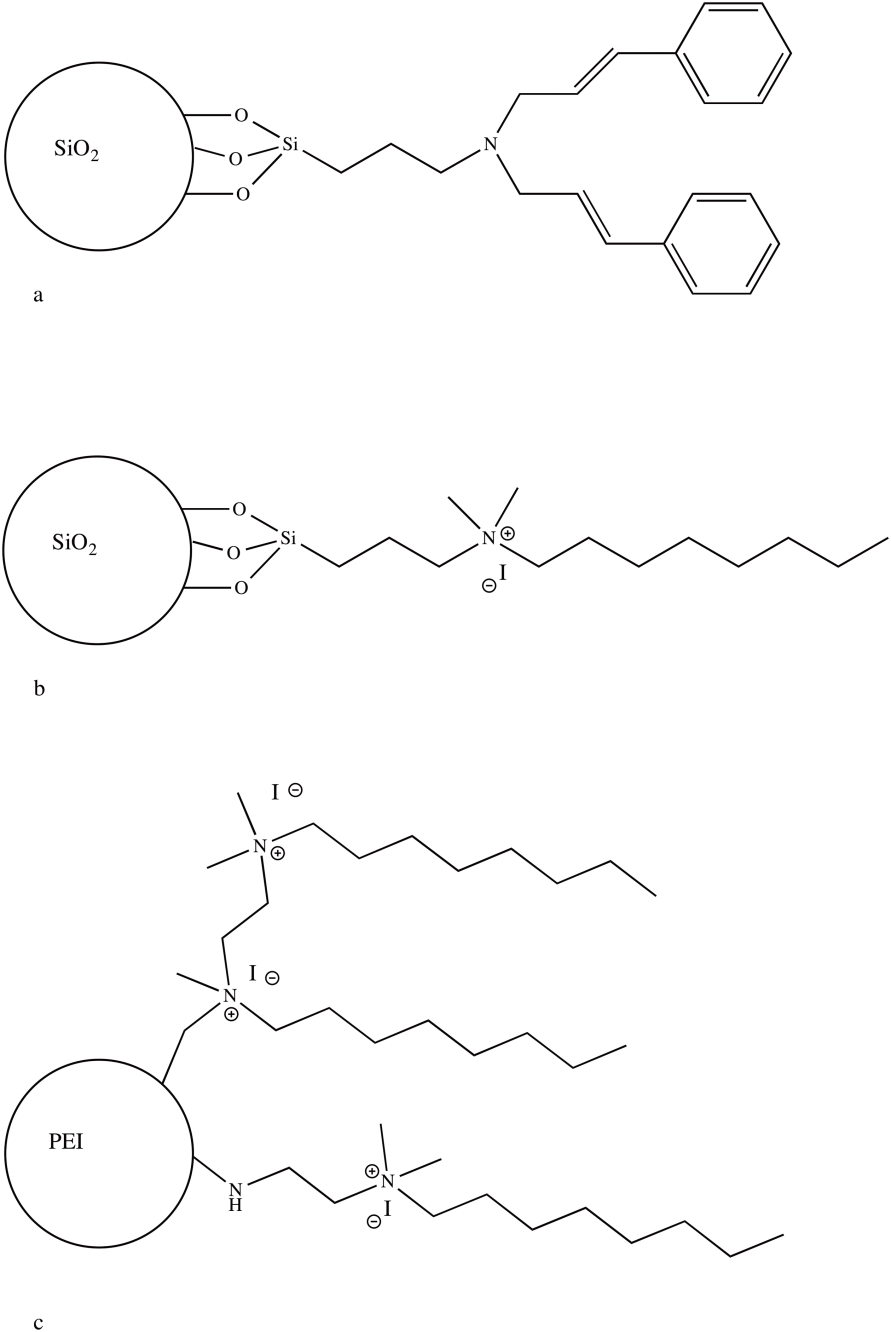
Structural formulas of antibacterial NPs. a–Silica particle with tertiary amine bearing two t-cial groups. b–Silica particle with dimethyl-octyl ammonium functional group. c–Quaternary ammonium polyethyleneimine particle.

Nanoscale silica dioxide NPs were prepared according to Stober [13]. In brief, 230 mL absolute ethanol (Merck, Darmstadt, Germany) and 20 mL 29% ammonia (Merck)) in water were mixed in a 500 ml round-bottom glass flask (RBF). A 10 mL volume of tetraethyl orthosilicate (TEOS) (Merck,) was added while stirring at room temperature with a magnet stirrer. After 3 hrs, the reaction mixture became opaque and the reaction was allowed to proceed for 24 hrs. The NPs were then recovered by centrifugation at 6000 rpm for 30 min and immersed in 20 mL absolute ethanol. This procedure was repeated five times to wash out all unreacted TEOS and ammonia, then dried under vacuum. Further surface modifications were performed to convert the obtained NPs into antibacterial filler. This was conducted once they were decorated with tertiary amine bearing two t-cial groups (Fig 1a) and, in another variant, with quaternary ammonium having one 8-carbon long chain and two methyl groups (Fig 1b).

### Functionalization of silica-core with t-cial NPs

A total 10 mL (0.043 mol) 3-aminopropyl triethoxysilane (APTES) (Merck) were dissolved in 50 mL toluene (Merck) in a 100 mL RBF. Then 2.2 equimolars (12 ml) of t-cial (Sigma-Aldrich, St. Louis, MO, USA) were added, followed by three drops of 98% sulphuric acid (Merck) to propagate imine formation. After stirring for 1hr with a magnetic stirrer at room temperature, dry NaBH_4_ (Acros Orgnics, Geel, Belgium) was added (4 equimolars), immediately followed by the addition of catalytic iodine (Merck,). The reaction was allowed to proceed for 24 hrs at 120°C under azeotropic distillation in a Dean-Stark apparatus. Next, the salts formed and unreacted NaBH_4_ were removed by gravitational filtration, and the solvent was evaporated under vacuum for 72 hrs. The chemical structure of APTES with t-cial (t-cial-APTES) was confirmed by mass spectrometry (Orbitrap Fusion Lumos Tribrid, Thermo Fisher Scientific, Waltham, MA), nuclear magnetic resonance (^1^H-NMR), (Varian 300-MHz, Varian inc. Palo Alto, CA), in DMSO-d_6_ (Sigma-Aldrich), and infrared spectroscopy (FT-IR) analysis (Nicolet i-10, Thermo Fisher Scientific, Waltham, MA) with an attenuated total reflectance (ATR) device.

The surface of silica NPs with t-cial-APTES was modified by suspending 1.0 g NPs in 10 mL toluene in the presence of 1 mL t-cial-APTES and a catalytic amount of glacial acetic acid. The suspension was stirred at room temperature for 24 hrs, then the particles were recovered by centrifugation/ethanol rinsing and stored under vacuum in the presence of dry silica gel. The degree of functionalization was determined with an elemental analyzer (2400/II CHN, Perkin Elmer, Waltham, MA) as the percentage of nitrogen (%N) and carbon (%C).

### Synthesis of silica-core functionalized with N-octyl, N,N-dimethyl ammonium NPs (QASi)

A total 10 mL (0.043 mol) APTES were dissolved in 50 mL anhydrous tetrahydrofuran (Sigma-Aldrich,) in 100 mL RBF. Then, to convert all the APTES primary amines into the *N*,*N*-dimethylamino form, 12.9 g (10 equimolars) of paraformaldehyde (Sigma-Aldrich) were added, immeditely followed by the addition of 10 mL formic acid (96%) (Sigma-Aldrich). The reductive amination reaction was conducted at room temperature and under continuous stirring for 24 hrs. Then the unreacted paraformaldehyde was removed by gravitational filtration and the solvent was evaporated under vacuum at 37°C for 72 hrs. Mass analysis was performed to confirm complete conversion of the primary amines into tertiary amines. N-octylation was performed with the Menchutkin reaction. A total 1.1 equimolar (8.5 mL) of 1-iodooctane (Sigma-Aldrich) in 100 mL absolute ethanol were added to *N*,*N*-dimethylamine-APTES. Alkylation was allowed to proceed at 60°C for 72 hrs. After most of the solvent evaporated under vacuum, the quaternary ammonium product was isolated by crystallization from diethyl ether.

### QPEI NPs synthesis

QPEI NPs were synthesized as previously described [11]. Briefly, 600,000–1,000,000 Da polyethyleneimine (Sigma-Aldrich) were lyophilized (10.1 g, 0.232 mol) and then dissolved in 100 mL absolute ethanol. PEI was crosslinked by reacting it with 0.04 equimolars 1,4-diiodopentane (Sigma-Aldrich) under reflux for 24 hrs. Next, 1 equimolars 1-iodooctane (Sigma-Aldrich) was added, followed by neutralization of the generated acid with NaHCO_3_ (Merck) Methylation was performed by the addition of 2.25 equimolmars iodomethane (Sigma-Aldrich) and 40°C for 72 hrs. The generated acid was neutralized with 1 equimolars NaHCO_3_ and the obtained NPs were recovered by the addition of double distilled water (DDW), resulting in particle precipitation. The QPEI NPs were rinsed again with DDW, lyophilized and pulverized to a fine powder. Infrared spectroscopy was used to benchmark the prepared QPEI NPs with those reported previously in the literature [11] (Fig 1c).

### Sample preparation

Acrylic resin-based, powder-liquid dental material, Unifast Trad, (GC Europe, Leuven, Belgium) served as host polymer to which the test NPs were added at 8% (wt/wt). We previously found that when QPEI nanoparticles are mixed with Unifast at 2, 4, 6, 8 and 16% (wt/wt), and screened with the direct contact test (DCT) against various bacteria, the optimal concentration is 8% (wt/wt) (data not shown). The NPs were first blended with the solid portion of the acrylic material, followed by the addition of the liquid portion according to the manufacturer’s instructions.

### Compressive strength

Cylindrical specimens of 0.4 mm diameter and 10 mm length, prepared in a polypropylene tube were allowed to polymerize at room temperature for 1 hr, then stored at 37°C for 24 hrs before testing. Each test group consisted of 10 specimens of polymerized resin material with 8% (wt/wt) NPs, specimens without NPs served as control. Compressive strength was determined with a universal testing machine (3366, Instron, Canton, MA) operated at a displacement speed of 1 mm/min. The data were analyzed immediately with Merlin software to calculate the compressive strength and the Young modulus.

### Degree of monomer conversion

Three samples for each test formulation, mixed according to the manufacturer’s specifications, were placed on the ATR surface of the FT-IR and allowed to polymerize. Infrared spectra were taken every 60sec. from the start of the reaction and characteristic absorption peaks in the infrared range for carbon-carbon double bonds (1638 cm^-1^) and reference peaks of carbon-oxygen double bonds (1720 cm^-1^) were recorded. The degree of monomer conversion (DC) was calculated according to the following equation:

$$\%DC=100\times (1-\frac{\frac{Ainitial(1638{cm}^{-1})}{Ainitioal(1720{cm}^{-1})}}{\frac{Afinal(1638{cm}^{-1})}{Afinal(1720{cm}^{-1})}}$$

where A is the light absorption at a defined wavenumber.

### Contact angle

Disc-shaped specimens (d = 5 mm, h = 2 mm) of Unifast material with 8% (wt/wt) NPs were prepared in a polypropylene mold, polymerized for 1 hr and then stored in DDW at 37°C for 24 hrs. Non-modified Unifast material discs served as control. All specimens were polished with No. 320 abrasive paper. The contact angle between the deionized water and the test samples was measured with a LAUDA tensiometer (FirsTen Angstroms, Portsmouth, VA). A 2 μL drop of DDW was applied 10 times to each surface. The contact angle was recorded as the average of 10 readings for each droplet, i.e. a total 100 contact angle reads from each material sample.

### Direct contact test (DCT)

Samples of resin material containing 8% (wt/wt) NPs were allowed to polymerize on the sidewalls of a polystyrene flat bottom 96-well plate (Nunclon, Nunc, Copenhagen, Denmark). After 7 days at 37°C, the samples were rinsed several times with sterile phosphate-buffered saline (PBS) to remove excess polymerized material and the plates were sterilized overnight under UV radiation.

The Gram-positive *Streptococus mutans*, *(strain* ATCC 35668), was cultured overnight in brain-heart infusion broth (BHI) (Merck) at 37°C. Then 100 μL from the upper layer were diluted with fresh BHI to an optical density (OD_650_) of 0.1 (˜10^6^ colony forming units /mL).

**Fig 2:**
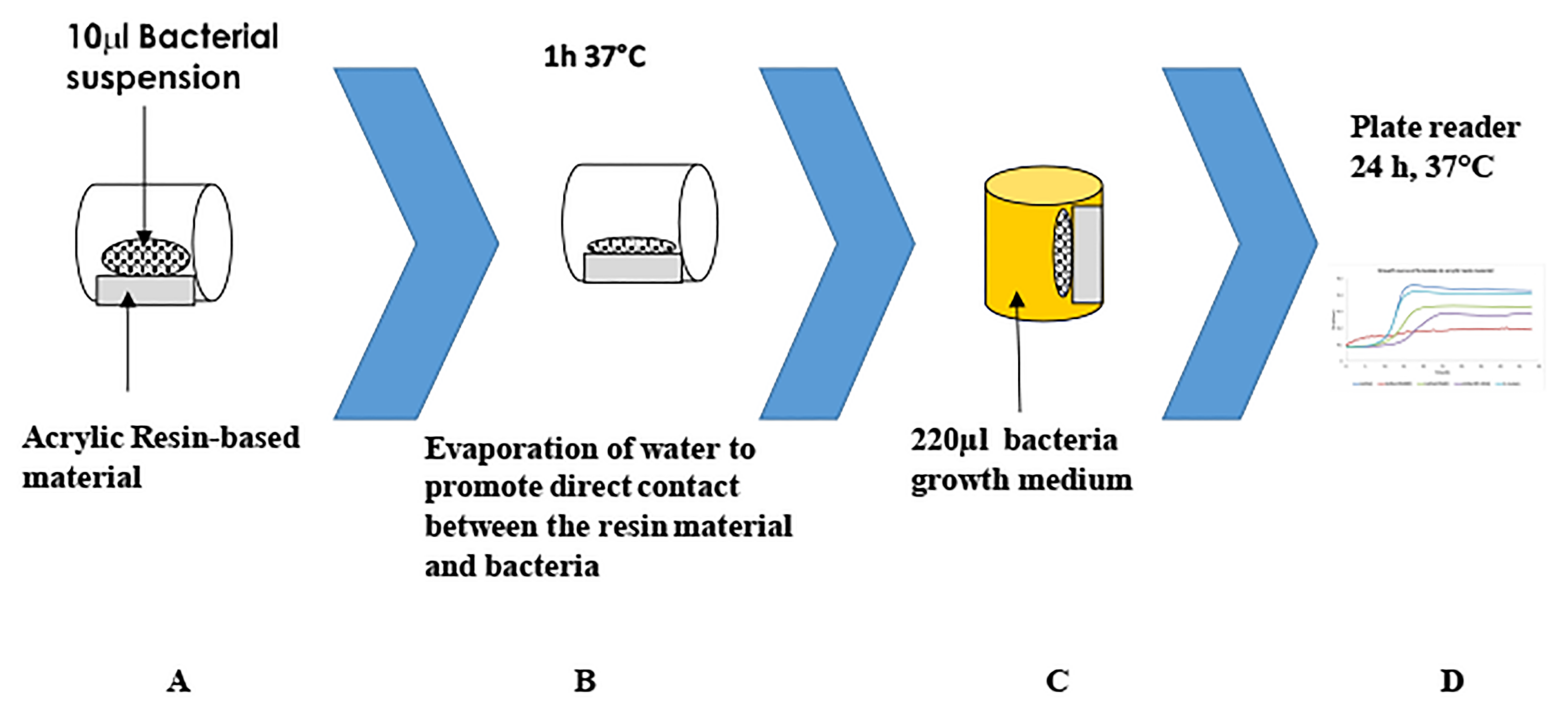
DCT procedure flowchart. A- a resin material samples allowed to polymerize onto microtite’s well sidewalls, followed by applying of bacteria suspension. B-Evaporation of suspension liquid. C- addition of growing media to agitate proliferation of survived bacteria. D- OD_650_ measured as indication for survived bacteria growth.

A flowchart with DCT procedure summarized in Fig 2. A total 10 μL bacterial suspension was placed on each sample surface and the plates were placed vertically and incubated at 37°C for ~40 min until all the liquid evaporated, to ensure physical contact between the tested surface and the bacteria. Then 220 μL BHI were added to each well and the plates were placed in a spectrophotometer (VERSAmax, Molecular Devices Corporation, Menlo Oaks Corporate Centre, Menlo Park, CA). OD_650_ measurements were taken every 20 min at 37°C during 24 hrs, and plotted as a function of time, representing bacterial growth curves, as previously described [27].

### Antibiofilm test under dynamic conditions

Biofilm formation was studied in a modified drip-flow reactor (MDFR), as described by Hahnel et al [28] and Ionescu et al [29] and consisted of three microbiological models:

1 - Nonospecific *S*. *mutans* biofilm grown for 48 hrs.
2- Same as above, but with prior simulation of salivary pellicle formation for 24 hrs;
3- Oral microcosm mixed plaque freshly pooled from human volunteers grown for 48 hrs with prior simulation of salivary pellicle formation for 24 hrs.

All the materials and reagents for the microbiological procedures were purchased from Sigma-Aldrich and all the culture media were purchased from Becton-Dickinson (BD Diagnostics-Difco, Franklin Lakes, NJ, USA).

**Fig 3:**
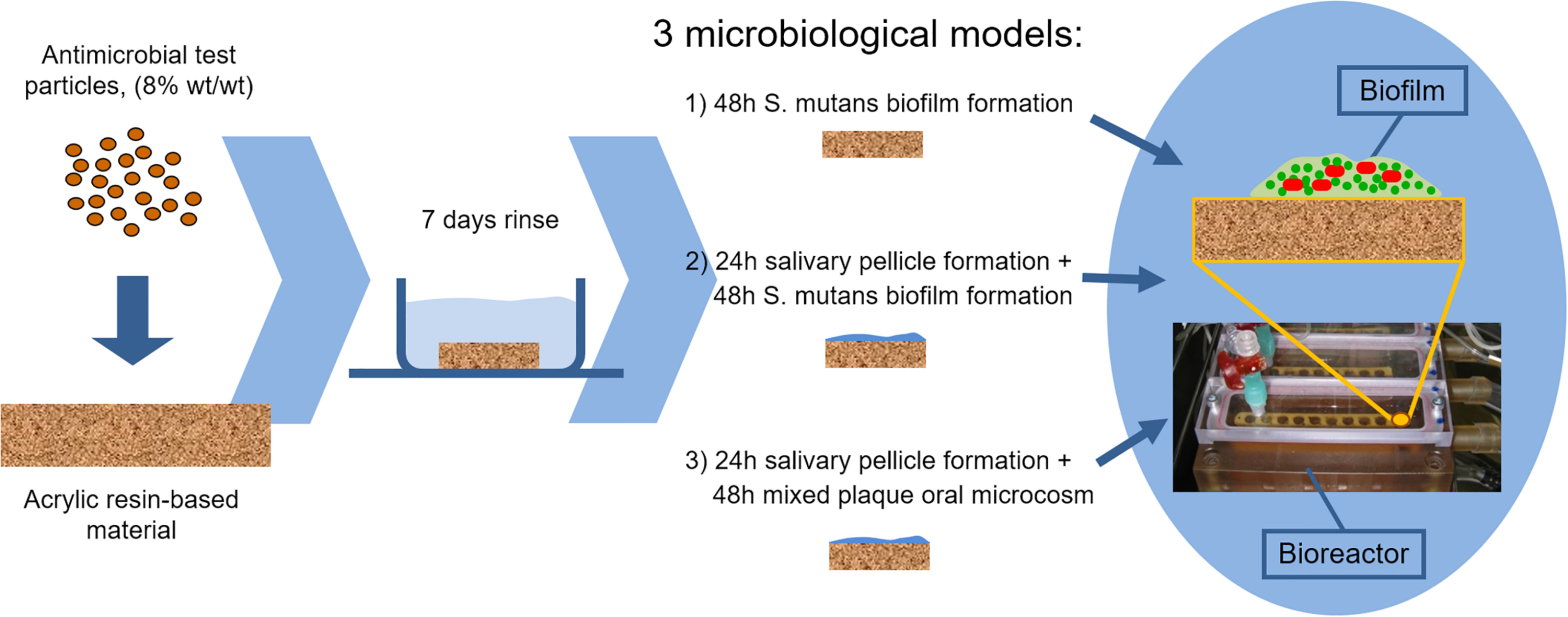
Antibiofilm test flowchart. Polymerized samples of modified resin material were first rinsed during 7 days. Then, samples of each one of the test groups were divided into 3 subgroups for three different biofilm models. The biofilm tests were performed using MDFR.

Fig 3 shows schematic presentation of antibiofilm tests. Specimens subjected to the anti-biofilm test were stored in 24-well plates under light-proof conditions and 1000 μL sterile PBS were added to each well. The plates were stored at room temperature for an additional 7 days to allow unreacted monomers or loosely attached antibacterial compounds to leach out of the specimens. To remove these compounds, each well was rinsed twice daily with 1000 μL sterile PBS.

### Saliva collection

Paraffin-stimulated whole saliva was collected by expectoration from three healthy donors (with their informed consent), in accordance with the protocol published by Guggenheim et al [30]. Briefly, saliva was collected in chilled test-tubes, pooled, heated at 60°C for 30 min to inactivate endogenous enzymes, and then centrifuged (12,000 x g) for 15 min at 4°C. The supernatant was transferred to sterile test-tubes, stored at -20°C and thawed at 37°C, 1 h before the experiments.

### Bacteria

A suspension of *S*. *mutans* strain ATCC 35668 in BHI broth was grown for 12 hrs at 37°C in an incubator with 5% CO_2_. The cells were harvested by centrifugation (2.200 rpm, 19°C, 5 min), washed twice with sterile PBS and re-suspended in fresh PBS buffer. The cell suspensions were sonicated (Sonifier model B-150; Branson, Danbury, CT, USA; operating at 7-W energy output for 30s) to disperse bacterial chains. The suspensions were then adjusted to OD_650_ = 1 on the McFarland scale, corresponding to ~3.0 x 10^8^ cells/ml.

### Modified drip-flow reactor

*B*iofilm formation was simulated under continuous flow conditions in a modified drip-flow reactor for 48 hrs [31]. Briefly, for simulating *S*. *mutans* biofilm formation under dynamic conditions, a modification of a commercially available drip-flow reactor (DFR 110, BioSurface Technologies; Bozeman, MT, USA) was used (MDFR). The modified design allows placement of customized sample carriers on the bottom of the flow cells, ensuring complete immersion of the specimens’ surfaces in the flow medium. The specimens were mounted on PTFE carriers, which were subsequently fixed on the bottom of each MDFR flow cell. Ten specimens for each test material were used, and the experiment was repeated three times. To minimize the risk of microbial contamination of the MDFR, all tubing and trays were sterilized in a low-temperature hydrogen peroxide gas plasma chemiclave (Sterrad, ASP; Irvine, CA) before assembly. The entire MDFR was then placed in a sterile hood.

In microbiological model 1, each cell was inoculated with 10 mL *S*. *mutans* suspension in early log phase to allow bacterial adhesion. After 4 hrs, a constant flow of sterile nutrient broth was provided with a multi-channel computer-controlled peristaltic pump (RP-1, Rainin; Emeryville, CA, USA). The broth was enriched with 10.0 g/L sucrose [32] and consisted of 2.5 g/L mucin (type II, porcine gastric), 2.0 g/L bacteriological peptone, 2.0 g/L tryptone, 1.0 g/L yeast extract, 0.35 g/L NaCl, 0.2 g/L KCl, 0.2 g/L CaCl2, 0.1 g/L cysteine hydrochloride, 0.001 g/L hemin, and 0.0002 g/L vitamin K1; the flow rate was set at 9.6 mL/hr and the temperature was maintained at 37°C [29].

In microbiological model 2, before inoculation the specimens’ surfaces were completely covered with thawed sterile saliva and maintained at 37°C for 24 hr. Then, after carefully aspirating and decanting the supernatant, 10 mL of *S*. *mutans* suspension in early log phase were inserted in each flow-cell to allow bacterial adhesion. After 4 hrs, a constant flow of nutrient broth was provided, as described above to allow bacterial adhesion and the MDFR continued to operate for 48 hrs.

In microbiological model 3, the specimens’ surfaces were completely covered with thawed sterile saliva and maintained at 37°C for 24 hrs to allow the formation of a salivary pellicle. Human whole saliva was collected by expectoration from at least three healthy volunteers who gave their informed consent to participate. The donors refrained from oral hygiene for 24 hrs, did not have any active dental disease, and had not consumed any antibiotics for at least the 3 months leading to the experiments. The saliva was collected within 30 min during a single session and the samples were pooled before further processing. Then the supernatant was gently discarded and 10 mL of pooled saliva were inoculated in each flow cell. After 4 hrs, to allow bacterial adhesion, a constant flow of nutrient broth was provided, and the MDFR continued to operate for 48 hrs, as described above.

### Viable biomass assessment

The viable biomass adhering to the specimen surfaces was assessed according to a tetrazolium salt (MTT) assay, as previously described [28]. Briefly, an MTT stock solution was prepared by dissolving 5 mg/mL 3-(4,5)-dimethylthiazol-2-yl-2,5-diphenyltetrazolium bromide in sterile PBS; a phenazinium salt (PMS) stock solution was prepared by dissolving 0.3 mg/mL N-methylphenazinium methyl sulphate in sterile PBS. The solutions were stored at 2°C in light-proof vials until the day of the experiment, when a fresh measurement solution (FMS) was prepared by diluting 1:10 v/v MTT stock solution and 1:10 v/v PMS stock solution in sterile PBS. A lysing solution (LS) was prepared by dissolving 10% v/v sodium dodecyl sulphate and 50% v/v dimethylformamide in distilled water.

After 48 hrs, the nutrient broth flow to the selected flow-cells of each microbiological model was discontinued. The specimen carriers were removed from the flow chambers and immediately placed in petri dishes containing sterile PBS at 37°C. Specimens were carefully detached from the tray with a pair of sterile tweezers, transferred to a plate containing sterile PBS at 37°C in order to remove non adherent streptococci, and placed in the wells of a sterile 48-well plate. A total 300μl FMS were added to each well, and the plates were incubated for 3 hrs at 37°C under light-proof conditions. During the incubation, electron transport across the microbial plasma membrane and, to a lesser extent, microbial redox systems converted the yellow MTT salt to insoluble purple formazan. The conversion was facilitated by the intermediate electron acceptor (PMS). The unreacted FMS was gently aspirated from the wells and the formazan crystals were then dissolved by adding 100 mL LS to each well and the plates were further incubated for 1 h at room temperature under lightproof conditions. Subsequently, 90 μL of the solution were transferred into the wells of 96-well plates. The absorbance of the supernatant was measured with a spectrophotometer (Genesis 10-S, Thermo Spectronic, Rochester, NY,) at a wavelength of 650 nm. The results, in OD_650_ units, were displayed graphically.

### Statistical procedures

Statistical analyses were performed with JMP 10.0 software (SAS Institute, Cary, NC, U.S.A.). Because of the low number of samples for some of the analyses, first the normality of distribution was checked and verified according to the Shapiro-Wilk test and hmogeneity of variances was checked and verified according to Levene’s test. One-way ANOVA and Tukey’s HSD post hoc test were used to highlight significant differences between experimental groups. The level of significance (α) was set at 0.05.

## Results

### Characterization of SiCial NPs

Mass spectroscopy revealed t-cial-APTES adduct peaks at 426.23 m/z (major) and 427.25 m/z (minor), compatible with a protonated cationic tertiary amine form. FT-IR spectra peaks were detected at 1027 cm^-1^ (Si-O), 1638 cm^-1^ (aromatic C = C), 1651 cm^-1^ (aliphatic C = C), 2851, 2920 cm^-1^ (-CH_2_-). The absence of peaks in the 3200–3600 cm^-1^ range confirmed the full conversion of primary and secondary amines to tertiary amines. H-NMR showed: 5.814 ppm (d, 2H, cis-aromatic N-CH = C), 7.546 ppm (t, 2H, aromatic), 7.516 ppm (d, 6H, aromatic), 7.568 ppm (d, 4H, aromatic).

Elemental analysis of functionalized NPs showed (SiCial): %N = 3.1, %C = 46, %H = 4.2. Average size: 165 ± 40 nm.

### Characterization of QASi NPs

FT-IR spectra peaks were: observed at 962 cm^-1^ (quaternary nitrogen) 1073 cm^-1^ (Si-O), 1638 cm^-1^ (aromaric C = C), 1389, 1443 cm^-1^ (C-H), 2851, 2926, 2972 cm^-1^ (-CH2-, CH_3_). There were no peaks in the 3200–3600 cm^-1^ range (primary and secondary amines). Elemental analysis of functionalized NPs (SiCial): %N = 3.87, %C = 33.6, %H = 4.2, %I = 22.9. Average size: 180 ± 56 nm.

### Characterization of QPEI NPs

FT-IR spectra peaks were observed at 962 cm^-1^ (quaternary nitrogen) 1460 cm^-1^ (primary amines N-H), 1638 cm^-1^ 1447 cm^-1^ (C-H), 2851, 2926, 2972 cm^-1^ (-CH2-, CH3-), 3431, 3460 cm^-1^ (primary and secondary amines N-H). Elemental analysis of functionalized NPs (SiCial):%N = 6.2, %C = 40.9, %H = 7.8, %I = 38.2 Average size: 12.0 ± 2.5 nm.

### Compressive strength

**Fig 4:**
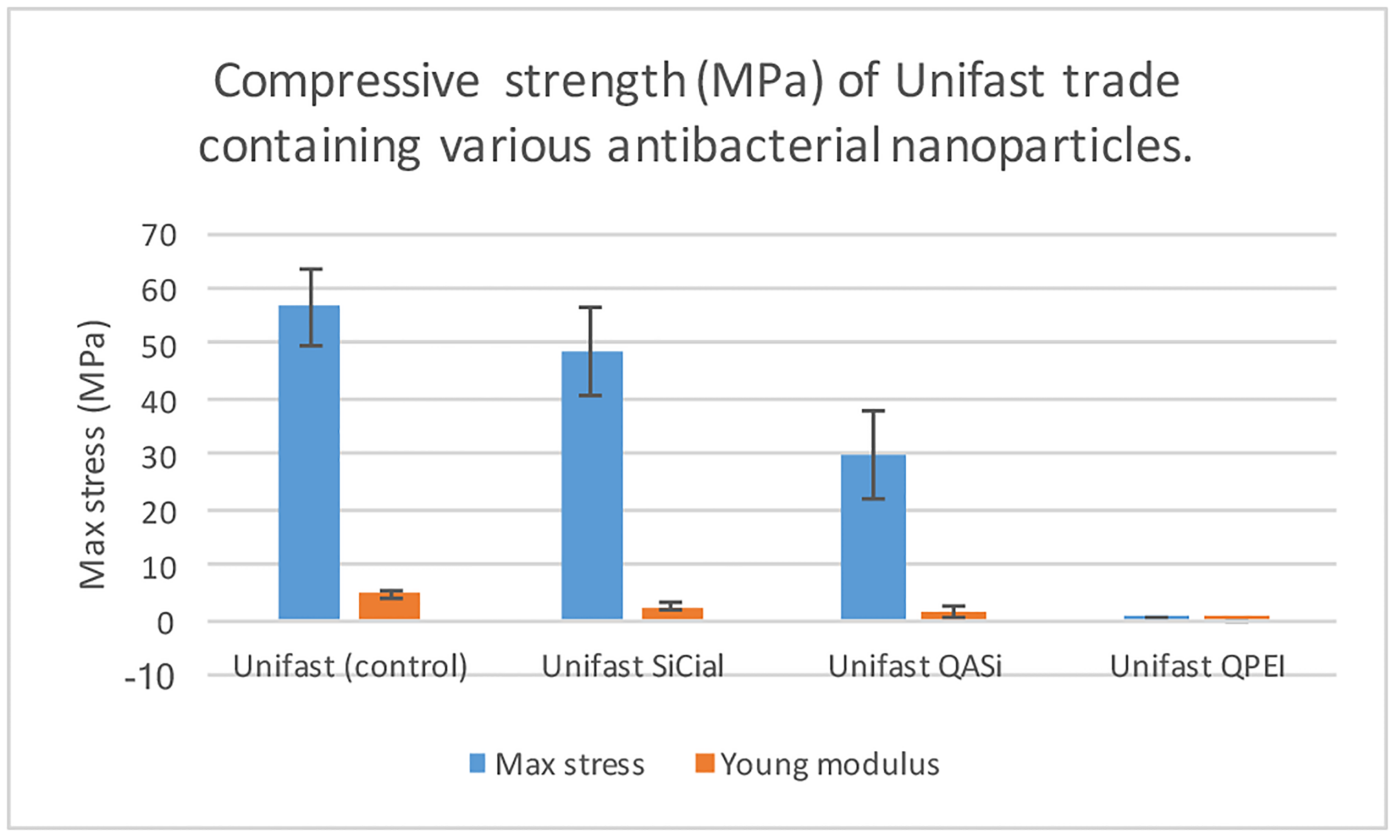
Comparison of maximal stress at rupture and the youngs modulus. Unmodified resin material served as control. As can be seen, the SiCial particles did not lead to a significant reduction in maximal stress or modulus. Addition of any of the quaternary ammonium-containing particles resulted in lower mechanical properties, QPEI particles causing complete destruction of the resin material.

Compressive strength and Young’s modulus are shown in Fig 4. A significantly strong decrease in mechanical strength was measured for Unifast samples with 8% (wt/wt) QASi, whereas the addition of the same amount of QPEI resulted in complete softening of the host material. The addition of 8% (wt/wt) SiCial caused only a slight decrease in compressive strength

### Degree of monomer conversion (DC)

**Fig 5:**
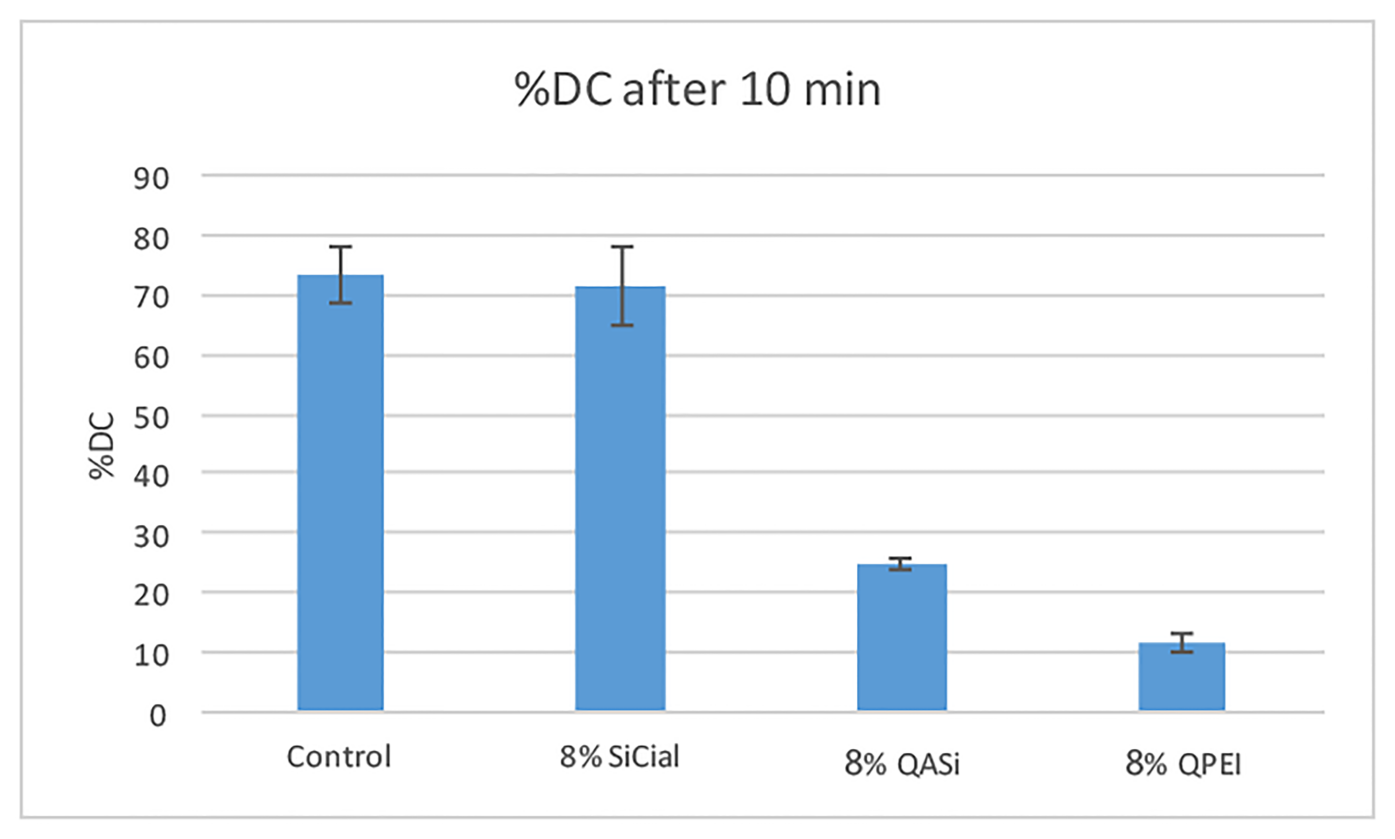
Degree of monomer conversion calculated from the data measured with infrared spectroscopy data. %DC values are expressed as mean with standard deviation. Quaternary ammonium iodide-containing particles caused a reduction in the degree of monomer conversion vs the base material. The SiCial particles preserved the %DC of the unmodified resin material.

**Fig 6:**
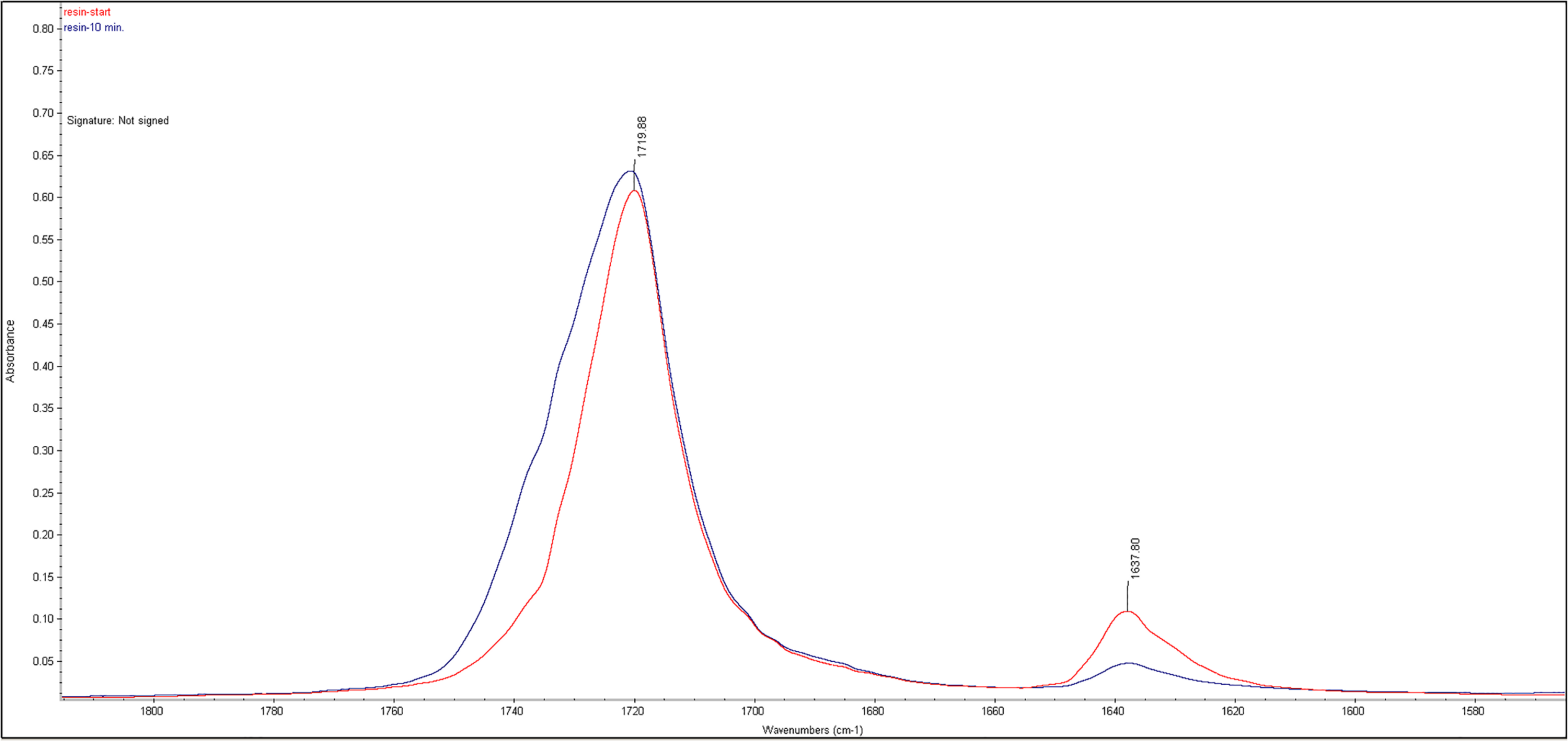
Representative FT-IR curves for DC calculation. Infrared spectra area focusing on characteristic peaks related to double carbon-carbon acrylic bonds (~1637nm) and carbon-oxygen double bond (~1720nm) used as normalization peaks of uncured resin material (red) and after 10 minutes of curing (blue).

The DC of the three test groups and of the control group are shown in Fig 5. Only a slight decrease in monomer conversion occurred in samples with incorporated SiCial NPs, whereas samples with quaternary ammonium functionality resulted in severe inhibition of polymerization. Moreover, the QPEI NPs terminated the polymerization at such an early stage, that the final material remained in soft solid phase. Fig 6 demonstrates representative FT-IR spectra of unmodified resin material before and after curing.

#### Discoloration

**Fig 7:**
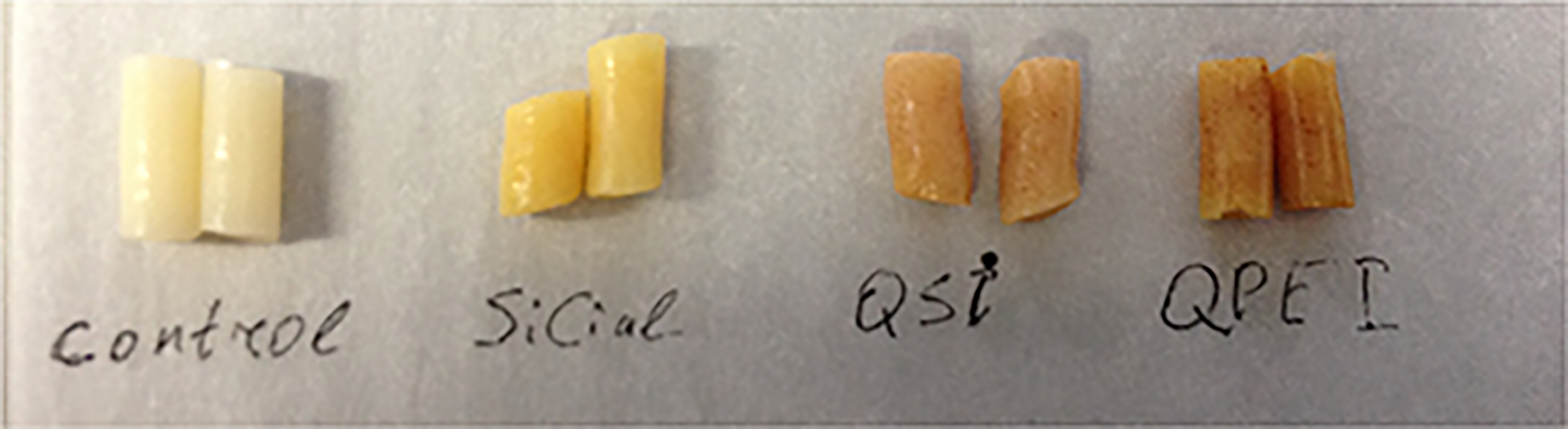
Color changes in resin material modified with antibacterial nanoparticles. Duplicates of all the test samples were compared with the unmodified control material. Changes in the color of the test samples are evident upon comparison of the unmodified material (control) with samples with incorporated quaternary ammonium iodide salt particles (i.e. QPEI and QASi). A relatively mild change in color is seen in samples with SiCial particles.

Fig 7 shows the significant discoloration in resin samples with incorporated nanoparticles containing quaternary ammonium iodide salts (QPEI and QASi) vs unmodified resin material (control). A relatively mild change in color is evident in samples with SiCial particles.

#### Contact angle

**Fig 8:**
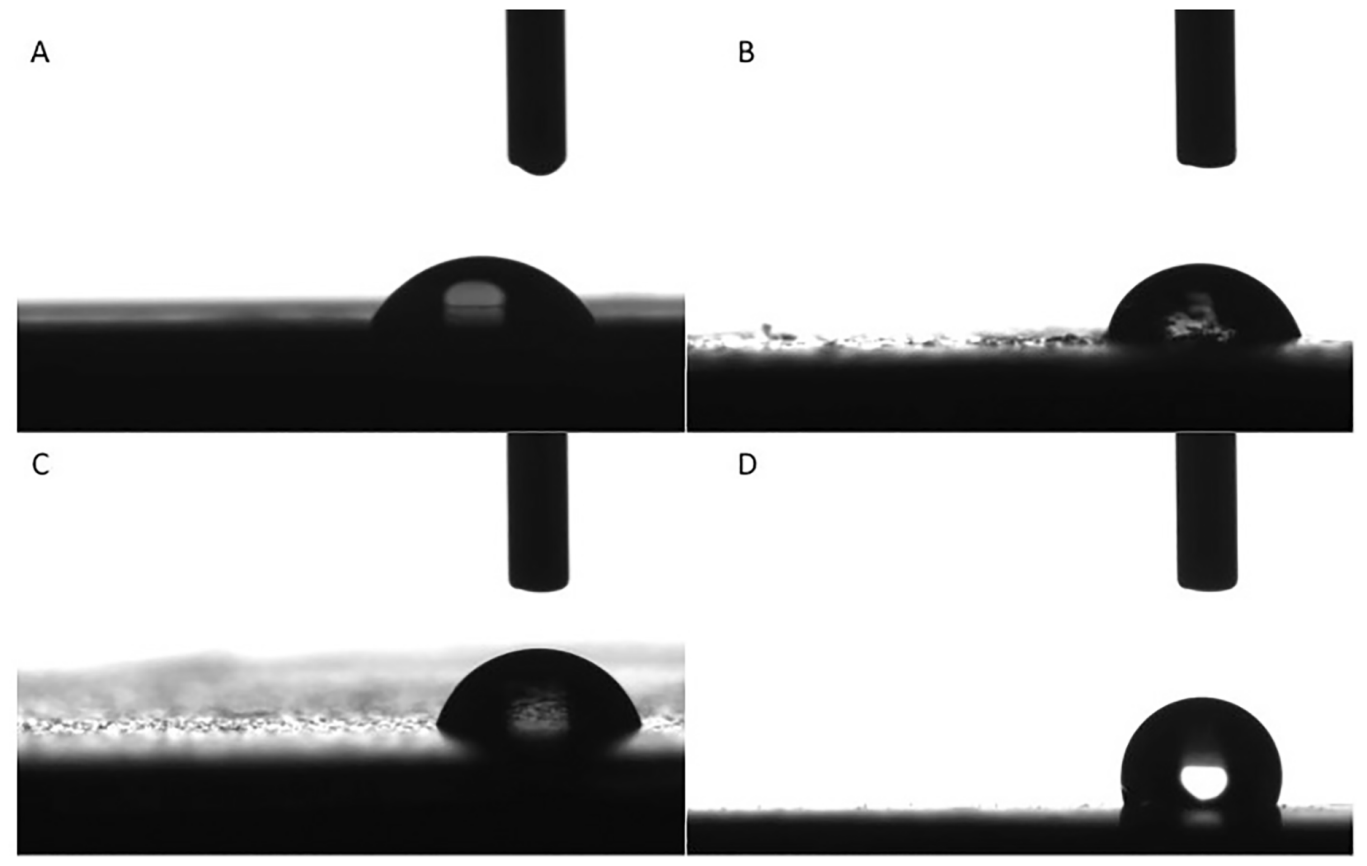
Contact angle measurements arranged in increasing order of hydrophobicity. A (unmodified, control): 59.4(±5.6)°; B (QPEI): 74.3(±5.1)°; C (QASi): 74.6(±1.9)° and D (SiCial): 111.2(±0.6)°.

As shown in Fig 8, the highest contact angle and thus highest hydrophobicity was observed for cinnamaldehyde-adduct NPs. Both QPEI and QASi NPs showed the same outer shell functionality and similar hydrophobicity. Unmodified acrylic polymer exhibited the highest hydrophilicity.

### Direct contact test

**Fig 9:**
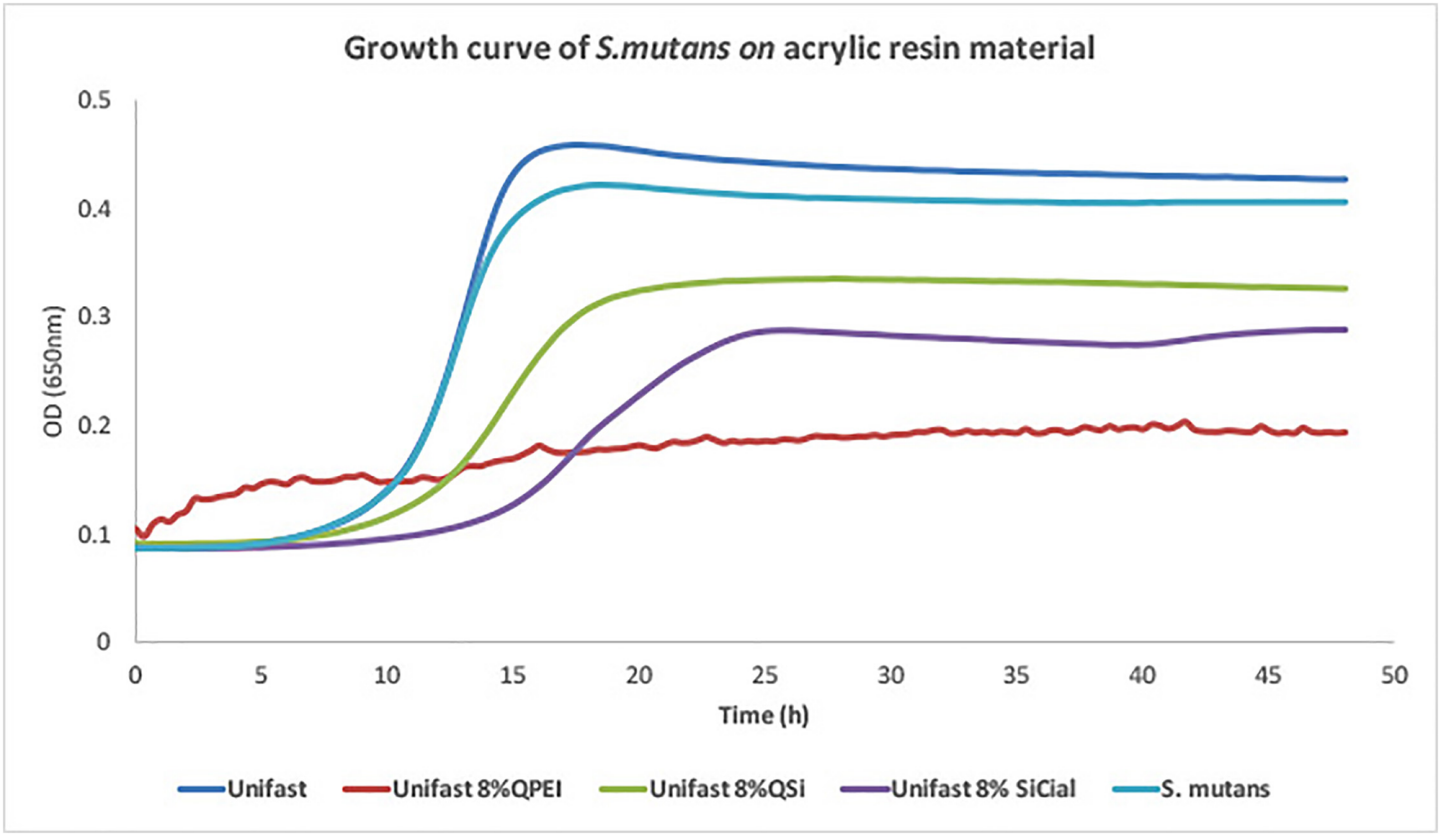
Antibacterial activity against *S*. *mutans* of unmodified acrylic material and with incorporated 8% QPEI, 8% QASi or 8% SiCial.

*S*. *mutans* growth curves during 48 hrs on acrylic resin material with the three antibacterial nanoparticles are shown in Fig 9. Samples containing 8% QPEI led to material disruption due to poor polymerization. This can be seen in the wavy nature of the related curve, resulting from fluctuations in optical density caused by material rupture and dissemination in the course of the experiment. Although samples of all the three test formulations resulted in some inhibition of bacteria growth, unmodified polymer had **no effect**. The QPEI samples showed the strongest antibacterial activity, followed by QASi and the lowest activity was observed for SiCial.

### Antibiofilm test

**Fig 10:**
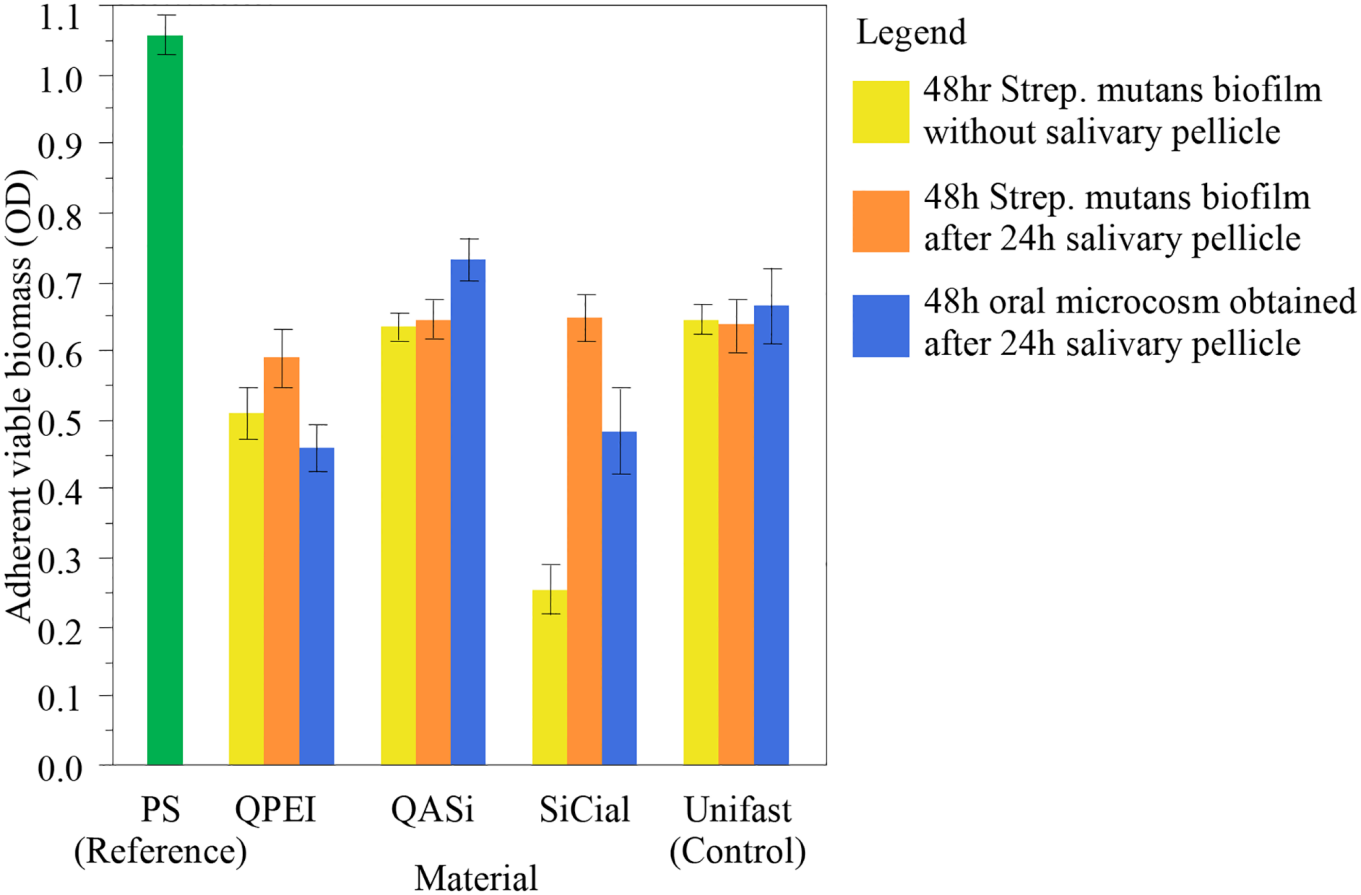
Antibiofilm effect. Each error bar was constructed according to 1 standard error from the mean OD values for each microbiological model. The different datasets were normalized for viable biofilm formation on the reference material,(polystyrene) of tissue-culture treated multiwall plates.

As the results of the statistical tests verified the assumptions of normality and homoscedasticity for all the microbiological data, it was possible to perform ANOVA and post-hoc tests. The biofilm data is depicted graphically in Fig 10; the statistical significance is shown in Table 1.

**Table 1:** Verification of assumptions of normality and homoscedasticity.

	Microbiological models		
Statistical assumptions	48 hr *S*. *mutans* without salivary pellicle	48 hr *S*. *mutans* after 24 hr salivary pellicle	48 hr oral microcosm after 24 hr salivary pellicle
**Normality (Shapiro-Wilk’s test)**	p = 0.0778	p = 0.1372	p = 0.6933
**Homoscedasticity (Levene’s test)**	p = 0.1557	p = 0.3265	p = 0.3486

p<0.05 rejects H_0_ (Shapiro-Wilk) that the data are from the normal distribution or (Levene) that the variances are equal.

The monospecific *S*. *mutans* microbiological model without simulation of salivary pellicle, showed significant antibacterial activity upon QPEI and SiCial incorporation. Biofilm formation was reduced by about 22% (p = 0.017) and 60% (p<0.001),). No significant differences in biofilm formation were found upon the addition of QASi.

Pre-treatment of specimen surfaces by simulating salivary pellicle formation for 24 hrs before allowing *S*. *mutans* biofilm formation resulted in a lack of antibacterial activity of all the experimental materials (Table 2, Fig 10).

**Table 2:** Biofilm formation on the surfaces of the test materials for each microbiological model.

	Microbiological models		
Materials	48 hrs *S*. *mutans* without salivary pellicle	48 hr *S*. *mutans* after 24 hr salivary pellicle	48 hr oral microcosm after 24 hr salivary pellicle
**Unifast + 8% QPEI**	0.510(0.118)^b^	0.590(0.103)^a^	0.458(0.083)^c^
**Unifast + 8% QASi**	0.635(0.048)^a,b^	0.646(0.067)^a^	0.733(0.077)^a^
**Unifast + 8% SiCial**	0.254(0.114)^c^	0.648(0.083)^a^	0.483(0.150)^b,c^
**Unifast (Control)**	0.646(0.069)^a^	0.636(0.093)^a^	0.665(0.134)^a,b^

The mean ODs are shown (+/- 1 SD). The different superscript letters (a, b) indicate statistically significant between group differences (Tukey’s test, p<0.05)

The microbiological model based on a mixed plaque oral microcosm biofilm grown after salivary pellicle formation for 24 hrs showed significant antibacterial activity of QPEI (31% reduction in biofilm formation, p = 0.027) and a borderline non-significant biofilm reduction by SiCial (27% reduction, p = 0.059).

## Discussion

The present study proposing two novel silica-core based NPs as a possible surface-active antibiofilm filler for resin-based dental materials, showed that SiCial was the most effective. IN comparison with the well-studied QPEI NPs, incorporation of SiCial NPs has a much lower antibiofilm effect, but it did not induce resin’s polymerization inhibition, the acrylic material preserving its original mechanical properties.

QPEI are well studied NPs that consist of a polyethyleneimine core and dimethyl-octyl ammonium iodide functional groups. The high activity of iodide anions in free-radical polymerization systems act in particular as peroxide activators in the generation of free radicals [12] [33]. As shown in Fig 5, this phenomenon was strongly reflected in the % DC measured for resin material with QPEI and QASi particles, both containing ammonium iodide. In contrast, resins containing SiCial NPs did not show a decrease in monomer conversion. The compressive strength test demonstrated a good correlation with the DC results as anticipated and shown in Fig 4

The hydrophobicity of resin samples modified with NPs vs. that of the unmodified material clearly demonstrate that the incorporation of 8%w of both polyethyleneimine-based and silica-based particles with dimethyl-octyl ammonium iodide functionality did not induce changing in the water contact angle (Fig 7). In contrast, di-cinnamyl amine increased the contact angle in a manner that changed the nature of the material’s surface from hydrophilic to hydrophobic. These findings may account for the reduced biofilm when tested without a salivary pellicle, i.e. fewer bacteria came in contact with the surface.

The DCT growth curves of *S*. *mutans* for test samples vs. unmodified resin material showed an only partial antibacterial effect for silica NPs but strong inhibition for QPEI (Fig 8). This is probably due to the lower loading degree of functional units attached to silica-core NPs. It is conceivable that the poor condition of the host resin material with the QPEI NPs resulted in material degradation, diffusion into the medium and thus an irregular curve. Both silica-based QASi and the SiCial NPs showed similarly mild results; the cinnamyl-modified nanoparticles were slightly more active than the quaternary ammonium ones This leads to the conclusion that immobilized t-cinnamaldehyde preserves its antibacterial potency after immobilization onto silica particle.

Recreating the complex oral environment in the lab is extremely challenging. The results are sound when interpreted within their known limits, and allow for isolation of several parameters under controlled conditions. Different microbiological models lead, therefore, to different results. It is clear that the salivary pellicle plays a prominent role as a moderator in the antibacterial activity of contact-active materials. The contact-active killing capacity of QPEI was previously described [3, 4, 7–10]. These particles, and the even more effective SiCial, were able to reduce biofilm formation of *S*. *mutans* biofilm after 48 hr in a continuous flow environment. However, the activity was hindered by the salivary pellicle, which probably acted as a separation layer between the bacterial cells and the contact-active surface, inactivating the materials’ antibacterial properties. It is conceivable, therefore, that the contact-killing feature of a dental material may be important during the first steps of adhesion and colonization, rather than after salivary pellicle formation, at least for early colonizing cocci such as *S*. *mutans*. The choice of incubation time in the present study allowed the formation of mature biofilm structures, such as those taking place in sites where cleaning by brushing is difficult to achieve. Our results suggest that under the test conditions the addition of QPEI nanoparticles results in reduced biofilm formation after 48 hrs and in the presence of a salivary pellicle. QPEI nanoparticles, being 10 times smaller than SiCial, may have enhanced activity, due to the smaller dimension and the consequent increase in surface to volume ratio. Indeed, the QPEI nanoparticles when incorporated in the carrier material resulted in a much less hydrophobic surface than the SiCial ones. A previous study demonstrated that resin with the higher hydrophobicity caused an increase in biofilm formation in the oral microcosm model vs the less hydrophobic one [29]. Our results indicate that the SiCial-adduct yields a hydrophobic surface, whereas QPEI and QASi NPs produce a slightly hydrophilic surface. For this reason, SiCial nanoparticles may be less efficient than QPEI as contact-killing agents on an oral microcosm biofilm. Further studies may help to assess whether the relative dimensions of the two nanoparticles or the resulting hydrophobicity of the material alone can account for their different antibacterial behavior. It bears mention that QASi NPs did not show any antibiofilm effect in the three microbiological biofilm models used. In view of all the above it maybe concluded that SiCial NPs although less efficient than QPEI NPs, are more effective as they do not destroy the resin material.
